# Cryo-EM structure of DNA-bound Smc5/6 reveals DNA clamping enabled by multi-subunit conformational changes

**DOI:** 10.1073/pnas.2202799119

**Published:** 2022-06-01

**Authors:** You Yu, Shibai Li, Zheng Ser, Huihui Kuang, Thane Than, Danying Guan, Xiaolan Zhao, Dinshaw J. Patel

**Affiliations:** ^a^Structural Biology Program, Memorial Sloan-Kettering Cancer Center, New York, NY, 10065;; ^b^Molecular Biology Program, Memorial Sloan-Kettering Cancer Center, New York, NY, 10065;; ^c^Functional Proteomics Laboratory, Institute of Molecular and Cell Biology, Agency for Science, Technology and Research (A*STAR), Singapore 138673, Singapore;; ^d^Simons Electron Microscopy Center, New York Structural Biology Center, New York, NY, 10027

**Keywords:** Smc5/6, DNA clamp, kleisin, KITE proteins, cryo-EM

## Abstract

Structural maintenance of chromosomes (SMC) complexes are essential for chromatin organization and functions throughout the cell cycle. The cohesin and condensin SMCs fold and tether DNA, while Smc5/6 directly promotes DNA replication and repair. The functions of SMCs rely on their abilities to engage DNA, but how Smc5/6 binds and translocates on DNA remains largely unknown. Here, we present a 3.8 Å cryogenic electron microscopy (cryo-EM) structure of DNA-bound *Saccharomyces cerevisiae* Smc5/6 complex containing five of its core subunits, including Smc5, Smc6, and the Nse1-3-4 subcomplex. Intricate interactions among these subunits support the formation of a clamp that encircles the DNA double helix. The positively charged inner surface of the clamp contacts DNA in a nonsequence-specific manner involving numerous DNA binding residues from four subunits. The DNA duplex is held up by Smc5 and 6 head regions and positioned between their coiled-coil arm regions, reflecting an engaged-head and open-arm configuration. The Nse3 subunit secures the DNA from above, while the hook-shaped Nse4 kleisin forms a scaffold connecting DNA and all other subunits. The Smc5/6 DNA clamp shares similarities with DNA-clamps formed by other SMCs but also exhibits differences that reflect its unique functions. Mapping cross-linking mass spectrometry data derived from DNA-free Smc5/6 to the DNA-bound Smc5/6 structure identifies multi-subunit conformational changes that enable DNA capture. Finally, mutational data from cells reveal distinct DNA binding contributions from each subunit to Smc5/6 chromatin association and cell fitness. In summary, our integrative study illuminates how a unique SMC complex engages DNA in supporting genome regulation.

## Significance

The Smc5/6 complex plays multiple roles in DNA replication and repair. Its genome-protecting functions rely on its interaction with DNA; however, how this complex engages DNA is poorly understood. We report on a cryogenic electron microscopy structure of DNA-bound budding yeast Smc5/6 complex, revealing that its subunits form a clamp to encircle a double-helical DNA. We define the multi-subunit interactions forming the DNA clamp and the DNA binding sites distributed among subunits. We identify subunit transformations upon DNA capture and functional effects conferred by its multiple DNA contact sites. Our findings, in conjunction with studies on other structural maintenance of chromosomes (SMC) complexes, suggest a common SMC DNA-clamp mechanism with individual complex specific features that enable diverse genome organization and protection functions by SMC family complexes.

Structural maintenance of chromosomes (SMC) complexes are essential genome regulators in both prokaryotes and eukaryotes. In eukaryotes, the cohesin and condensin SMC complexes organize DNA, while the Smc5/6 complex (referred to as Smc5/6) directly regulates DNA replication and repair (1). At the structural level, SMC complexes share similarities while possessing unique attributes (1). Each complex contains a pair of SMC subunits and a set of non-SMC subunits. The SMC subunits define the tripartite filamentous architecture of the complex: their approximal 50-nm long coiled coil arm region connects their dimerized hinge and adenosine triphosphatase (ATPase) head regions (1). A non-SMC kleisin subunit uses its *N*- and C-terminal domains to link the head of one SMC to the head-proximal arm region (neck) of another SMC, forming a trimeric SMC-kleisin structure. In cohesin and condensin, two large U-shaped HEAT (Huntington, elongation factor 3, PR65/A, TOR) repeat HAWK (HEAT proteins associated with kleisins) subunits attach to the middle region of the kleisin. By contrast, the Smc5/6 kleisin (Nse4) binds to smaller WH (winged helix)-containing KITE (kleisin interacting tandem WH elements) subunits (Nse1 and Nse3) (2).

SMC-mediated functions depend on interactions with DNA. Recent cryogenic electron microscopy (cryo-EM) structures of DNA-bound cohesin and condensin revealed that their HAWK subunits and the SMC head-neck regions form a clamp to enclose a single DNA double helix (3–7). DNA clamping can be critical for cohesin and condensin to extrude DNA loops for chromatin folding (5, 7–9). DNA loop extrusion additionally requires arm bending at a region called the elbow, which is found in both cohesin and condensin (5, 7–9). By contrast, a lack of arm bending in Smc5/6 was suggested by negative stain EM and cross-linking mass spectrometry (CLMS) data (10–14). Since Smc5/6 does not contain HAWK proteins nor shows arm-bending, it has remained unclear how Smc5/6 engages DNA to accomplish its multiple functions.

Here we address the molecular mechanisms by which this unique SMC complex binds DNA using an integrative approach, coupling a cryo-EM-based structural characterization with CLMS analyses and functional investigation. Our atomic structure of a DNA-bound *Saccharomyces cerevisiae* Smc5/6 complex reveals that the head-neck Smc5-6 regions and the Nse1-3-4 subcomplex together form a clamp entrapping the DNA helix. The structure further reveals protein subunit folds and association, as well as how the subunits collaborate to entrap DNA. Comparison of CLMS analyses of DNA-free Smc5/6 with the structure of the DNA-bound Smc5/6 unveils large scale, multi-subunit conformational changes that enable Smc5/6 to encircle DNA. Finally, our mutational data suggest distinct contributions from each of the DNA binding regions to Smc5/6 chromatin association and cellular fitness. Comparison of our findings with those of other SMCs reveals that diverse SMC complexes use a similar DNA clamping strategy despite structural differences, and that Smc5/6 possesses unique features distinct from cohesin, condensin, and prokaryotic SMCs. Our work lays the foundation for an in-depth understanding of how Smc5/6 fulfills unique roles in genome protection.

## Results

### Cryo-EM structure of DNA-bound Smc5/6 complex.

**Fig. 1.**
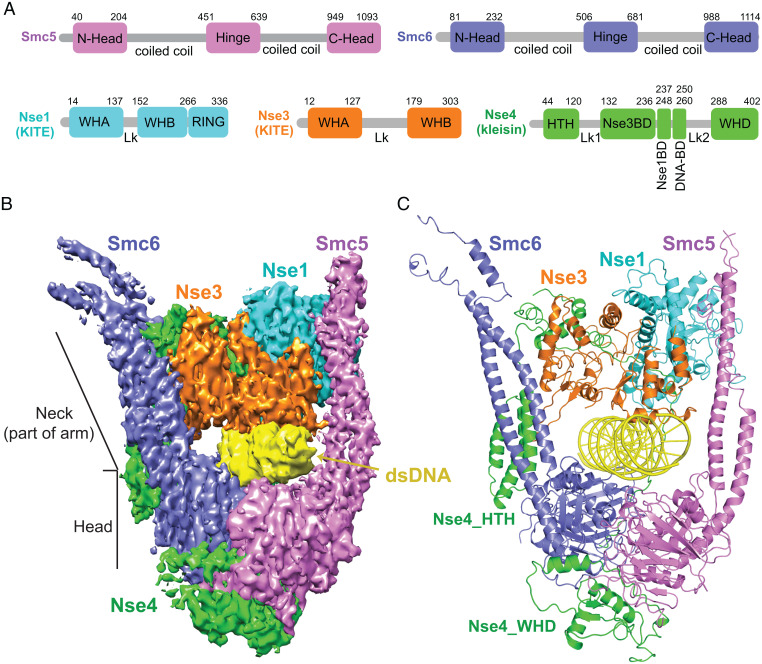
Cryo-EM structure of the DNA-bound *S. cerevisiae* Smc5/6 complex. (*A*) Domain organization of Smc5, Smc6 and the Nse1, Nse3, and Nse4 subunits. (*B* and *C*) 3.8 Å cryo-EM structure of DNA-bound Smc5/6 complex in an electron density representation (*B*) and in a ribbon representation (*C*). Proteins are color-coded as in panel A with dsDNA in yellow.

For structural insight into how Smc5/6 engages with DNA, we focused on the budding yeast complex that contains five DNA binding subunits (Smc5, Smc6, and Nse1-3-4) (Fig. 1*A*) and the Nse2 subunit that stabilizes Smc5 by associating with its midarm region (15, 16). This hexameric version of the complex can entrap DNA and its activity is enhanced by mutating the Walker B motif of Smc5 and Smc6 (Smc5-E1015Q, Smc6-E1048Q, or Smc5/6-EQ) to slow ATP hydrolysis (11). We purified this Smc5/6-EQ hexameric complex (referred to hereafter as Smc5/6 for simplicity) (*SI Appendix*, Fig. S1) using an expression system published and kindly provided by the Stephan Gruber laboratory (11). The complex was mixed with 72-bp dsDNA in the presence of ATP and Mg^2+^ before proceeding to cryo-EM grid preparation and data collection.

Three-dimensional (3D) reconstruction from cryo-EM particles (*SI Appendix*, Figs. S2 and S3*A*; and Table S1) allowed us to generate an atomic structure of DNA-bound Smc5/6 (Fig. 1 *B* and *C*). The structures of the head and neck regions of Smc5 and Smc6, the Nse1, Nse3 and Nse4 subunits, and 27-bp of bound DNA were determined at 3.8 Å resolution (Fig. 1 *A*–*C*, *SI Appendix*, Fig. S3 *B* and *C*), whereas other parts of the complex were not visualized due to flexibility (*SI Appendix*, Fig. S2*D*). While the DNA double helix could be placed into its density, it could only be modeled as an oligoA-oligoT duplex due to inability to definitively identify individual bases. In this structure, a single DNA double helix is encircled inside a clamp formed by the Smc5 and 6 head and neck regions in conjunction with the Nse1-3-4 subcomplex. We describe below how the DNA clamp is formed and how DNA is stabilized in the interior of the clamp. We also highlight the common and distinct features of Smc5/6 compared with DNA-bound structures of cohesin (3–5), condensin (6, 7), and the prokaryotic MukBEF SMC complex (17).

### Multi-subunit interactions support DNA clamp formation.

**Fig. 2.**
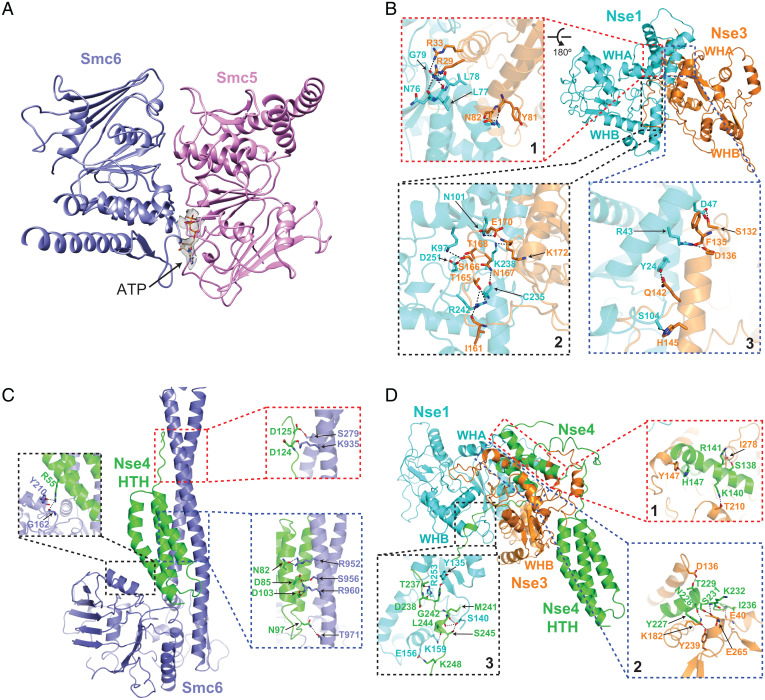
Subunit interactions in the structure of DNA-bound Smc5/6 complex. (*A*) The head domains of Smc5 and Smc6 adopt an engaged alignment in the complex. We observed density for ATP in the ATP-binding pocket of Smc5 head domain while ATP was not traceable in the Smc6 pocket. (*B*) Intermolecular hydrogen bonding contacts between Nse1 and Nse3 spanning three interfaces. (*C*) Intermolecular contacts between the HTH domain of Nse4 and the coiled-coil neck region of Smc6. (*D*) Intermolecular contacts between the midregion of Nse4 with Nse1 and Nse3. Details of hydrogen bonding interactions are shown in expanded Boxes 1 to 3 in *B* to *D*.

In the DNA-bound Smc5/6 structure, the two head regions interact with one another in an engaged form (E-head) (Fig. 2*A*). This is similar to the engaged cohesin head structures wherein two ATP molecules bridge head dimerization (*SI Appendix*, Fig. S3*D*), but different from the juxtaposed alignment of two head domains seen in DNA-free condensin (*SI Appendix*, Fig. S3*E*) (4, 18). The E-head of Smc5 and 6 constitutes the bottom part of the DNA clamp and interacts with two turns of the DNA double helix (Fig. 1 *B* and *C*). The Smc5 and 6 neck regions emanate upwards from the E-head domains with an approximate 35° tilt relative to the enclosed DNA and delineate the sides of the clamp without directly contacting DNA (Fig. 1 *B* and *C* and *SI Appendix*, Fig. S3*A*). A similar tilt is seen for cohesin and condensin, but not the prokaryotic SMC complex MukBEF (3–7, 17).

The top portion of the DNA clamp is formed by the interacting Nse1 and Nse3 KITE subunits that are oriented toward the neck regions of Smc5 and Smc6, respectively (Fig. 1 *B* and *C*). Our structural data support folding predictions that yeast Nse1 and Nse3 each contains two WH domains (WHA and WHB) connected by a linker (Lk), while Nse1 additionally possesses a C-terminal RING (really interesting new gene) domain positioned adjacent to the Smc5 neck region (Fig. 1 *A*–*C*) (2). Similar to their human and *Xenopus* counterparts, Nse1 and 3 interact through their WHA domains (Fig. 2*B*, Box 1) (19, 20). Another interface is formed between the Nse1-WHA/WHB and the Nse3 linker (Fig. 2*B*, Box 2), reminiscent of a *Xenopus* Nse1-3 interface formed only when bound to a Nse4 peptide, suggesting a universal role for Nse4 in stabilizing the KITE pair association (19). The current structure of DNA-bound Smc5/6 further reveals a third Nse1-3 interface specific for the yeast proteins (Fig. 2*B*, Box 3), whereby a yeast-specific region within the Nse3 linker contacts multiple residues in Nse1 WHA domain. As such, yeast Nse1 and 3 has a larger inter-KITE interface, by extension a more stable KITE-KITE association, when compared to their metazoan counterparts.

**Fig. 3.**
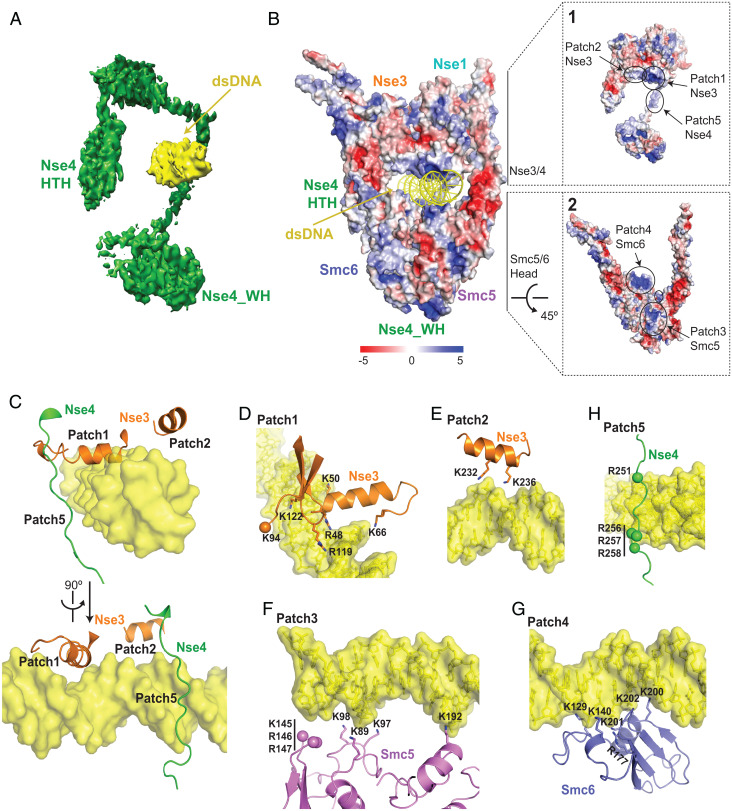
Protein-DNA contacts in the structure of the DNA-bound Smc5/6 complex. (*A*) The trajectory of the hook-shaped fold of Nse4 relative to DNA. (*B*) Electrostatic representation of DNA-bound Smc5/6 subunits. Expanded Boxes 1 and 2 outline the electrostatics of Nse3-4 and Smc5-6 subunits, respectively. (*C*) Two views rotated by 90° showing protein-DNA interactions involving the helical elements of Nse3 (patches 1 and 2) and the Nse4-DB region (patch 5). (*D to H*) Details of protein-DNA contacts associated with patch 1 (*D*), patch 2 (*E*), patch 3 (*F*), patch 4 (*G*) and patch 5 (*H*). Observable densities for Lys and Arg side chains are shown in a ribbon representation while side chain densities that could not be traced are shown as balls.

Connecting all parts of the structure is the hook shaped Nse4 kleisin (Fig. 3*A*). We were able to trace close to 80% of the Nse4 protein in the DNA-bound Smc5/6 structure, thus providing a comprehensive view of this elongated subunit. The N-terminal helix-turn-helix (HTH) of Nse4 interacts with the Smc6 neck (Fig. 2*C*), while its C-terminal WH domain (WHD) binds underneath the Smc5 head (Fig. 1 *B* and *C*). This behavior is similar to other kleisins, confirming a conserved kleisin-SMC interaction mode across diverse SMC complexes (1).

We further identify three roles for the midsection of Nse4. First, Nse4 contains an Nse3-binding domain (Nse3-BD) featuring two helices that interact with Nse3 (Fig. 2*D*, Boxes 1 and 2). While a similar situation was seen for *Xenopus* Nse1-3 bound to a Nse4 peptide, the yeast Nse3 contains a large insertion (*SI Appendix*, Fig. S4*A*) that may serve a regulatory role (19). Despite this difference, the structure of the *Xenopus* Nse1-3 bound to Nse4 superposes well with the yeast Nse1-3-4 structure in the DNA-bound Smc5/6 with an r.m.s.d. of 2.7 Å (*SI Appendix*, Fig. S4*B*). Second, a short segment of Nse4 (Nse1-BD) binds to Nse1 through an area formed by residues spanning all its three domains (Fig. 2*D*, Box 3). Third, an extended loop region of Nse4 (DNA-BD) runs along the minor grove of dsDNA (Fig. 3*A*). The interactions described above enable Nse4 to link four other subunits of the complex with DNA to aid DNA clamp formation.

### DNA-binding inner layer of the Smc5/6 clamp sculpted by four subunits.

We observed 20-bp of DNA entrapped in a central tunnel formed by Smc5 and 6 together with Nse3 and 4 (Fig. 1 *B* and *C*). Basic side chains from two dozen positively charged residues contributed by the four subunits line up on the inner face of the central tunnel and form contacts with the DNA backbone (*SI Appendix*, Fig. S5 *A* and *B*). Such sequence-nonspecific binding can permit genome surveillance with no sequence restrictions. The electrostatic surface representation of the complex reveals five distinct DNA binding patches (Fig. 3*B*, Boxes 1 and 2). Nse3 is solely responsible for binding to the top of the DNA: its WHA deploys an alpha-helix and adjacent loop (Patch 1) to associate along the major groove of DNA, while the adjacent DNA is secured by another helix from its WHB domain (Patch 2) (Fig. 3 *B*–*E*). Altogether, the side chains of eight lysine and arginine residues in Nse3 stabilize DNA association (Fig. 3 *D* and *E* and *SI Appendix*, Fig. S5*A*). Corresponding residues for a subset of these in the metazoan Nse3 proteins have been noted to affect in vitro DNA binding (19, 21), suggesting a partially conserved Nse3-DNA interaction mechanism from yeast to animals.

At the opposite side from Nse3, DNA is held up by the Smc5 and Smc6 head regions (Fig. 3*B*). Smc5 contributes seven (Patch 3, Fig. 3*F*) while Smc6 contributes six (Patch 4, Fig. 3*G*) lysine and arginine residues for DNA binding (*SI Appendix*, Fig. S5*B*). These are all located within the *N*-lobe of the head region in a similar fashion as the DNA binding sites in other SMCs, suggesting that SMC head-DNA binding is conserved across SMC family members (3–7, 17). However, unlike cohesin, condensin and MukBEF, the neck regions of Smc5/6 do not contact DNA (Fig. 1 *B* and *C*) (3–7, 17). Correlating with this, DNA and the neck region of Smc5/6 are separated by space (Fig. 1 *B* and *C*), which in principle could help accommodate damaged or structurally altered DNA for the DNA repair functions noted specifically for Smc5/6. Finally, the Nse4 DNA-BD provides four arginine side chains to interact with and nudge the DNA from its side (Fig. 3 *B*, *C*, and *H*). Notably, the Nse4-DNA association differs from the kleisin HTH-DNA association seen in cohesin and condensin (3–7).

### DNA binding sites from four subunits have different functional contributions.

We moved on to examine the cellular effects of DNA association contributed by each of the four subunits of Smc5/6. To this end, we used gene replacement to generate four mutants affecting each subunit’s DNA binding residue. These include *nse3^DNAm^* (*R48, K50, K66, K94, R119, K122, K232, K236 all to A*) mutated for DNA binding residues in Patches 1 and 2 (Fig. 3 *D* and *E*), *nse4^DNAm^* (*R251, R256, R257, R258 all to E*) mutated for those in Patch 5 (Fig. 3*H*), *smc5^DNAm^* (*K89, K97, K98, K145, R146, R147, K192 all to A*) mutated for residues in Patch 3 (Fig. 3*F*), and *smc6^DNAm^* (*K129, K140, R177, K200, K201, K202 all to A*) mutated for residues in Patch 4 (Fig. 3*G*). Each mutant protein was fused with a Flag tag module to monitor protein behavior. The tag did not interfere with protein function, as wild-type protein containing the same tag supported normal growth in all cases (below). As DNA binding is key to Smc5/6-mediated functions, perturbing this activity likely affect cell growth. We thus generated DNA binding mutants in diploid yeasts wherein only one of the two copies of the genes was mutated. The resultant diploid cells were then sporulated to give rise to haploid spore clones containing either the wild-type or the mutant allele.

**Fig. 4.**
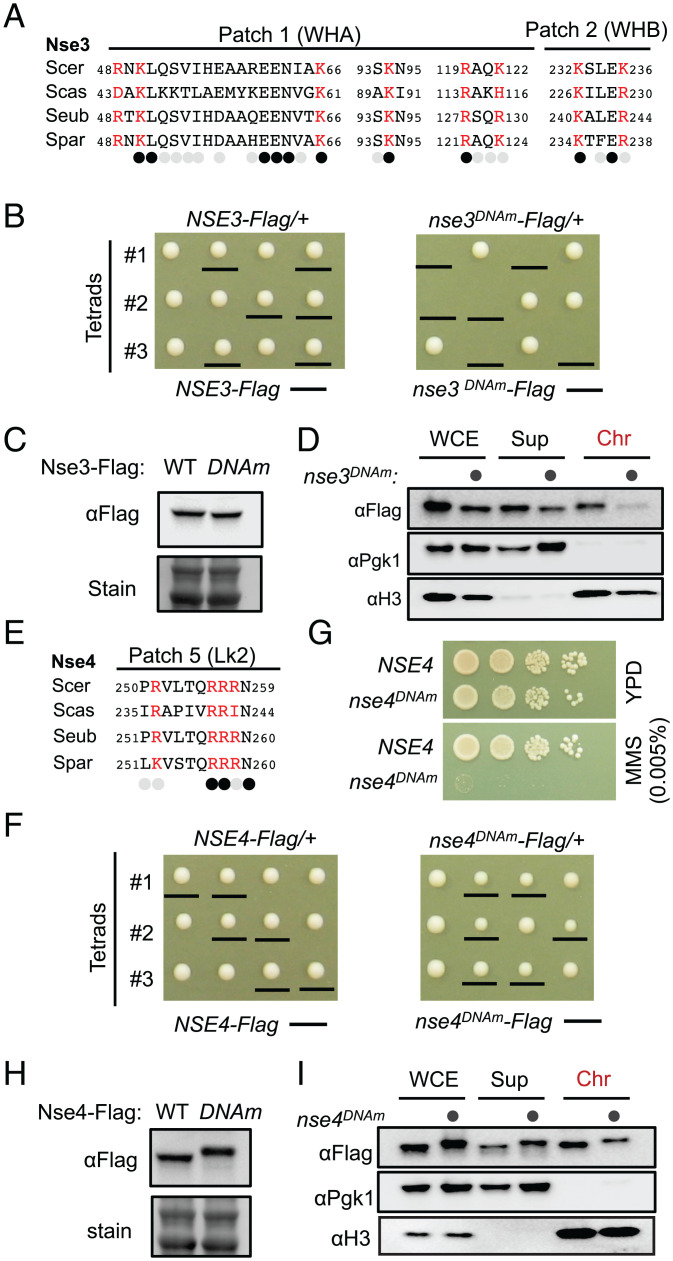
Assessment of the mutations affecting the DNA binding sites on Nse3 and Nse4. (*A* and *E*) Sequence alignment of Nse3 (*A*) or Nse4 (*E*) DNA-binding regions shows that their DNA binding sites (red) are composed of mostly conserved residues. *Saccharomyces* species examined include *S. cerevisiae* (*Scer), Saccharomyces castellii* (*Scas*), *Saccharomyces eubayanus (Seub*), and *Saccharomyces paradoxus* (*Spar*). Identical and similar amino acids are indicated by black and gray circles, respectively, while nonconserved amino acids are not labeled. (*B* and *F*) The *nse3^DNAm^* allele leads to cell unviability (*B*), while the *nse4^DNAm^* allele leads to slow growth (*F*). (*C, H*) DNA binding site mutations in Nse3 and Nse4 do not affect their protein levels. The shift of the Nse4 mutant protein band is likely due to increased numbers of negatively charged residues. (*D* and *I*) The effects of DNA binding site mutations in Nse3 (*D*) and Nse4 (*I*) on their chromatin association. Histone H3 and Pgk1 were used as markers for chromatin and nonchromatin fractions, respectively. (*G*) *nse4^DNAm^* causes DNA damage sensitivity.

First, we found that the *nse3^DNAm^* haploid cells were inviable, while the control cells containing tagged wild-type Nse3 gave rise to normal spore clones (Fig. 4 *A* and *B*). When the heterozygous diploid cells were examined, Nse3^DNAm^ and Nse3^WT^ showed similar protein levels (Fig. 4*C*), but chromatin bound Nse3^DNAm^ level was largely reduced (Fig. 4*D*). These results suggest that Nse3 DNA binding ability correlates with its chromatin association and cell viability.

By contrast to *nse3^DNAm^*, *nse4^DNAm^* spore clones were viable albeit slow growing (Fig. 4 *E* and *F*) and strongly sensitive to the DNA methylation agent MMS (methyl methanesulfonate) (Fig. 4*G*) The levels of total Nse4 protein were not changed by *nse4^DNAm^* (Fig. 4*H*), but those associated with chromatin were moderately reduced (Fig. 4*I*). Thus, Nse4-DNA binding sites contribute to its chromatin association and are critical for genotoxic resistance.

**Fig. 5.**
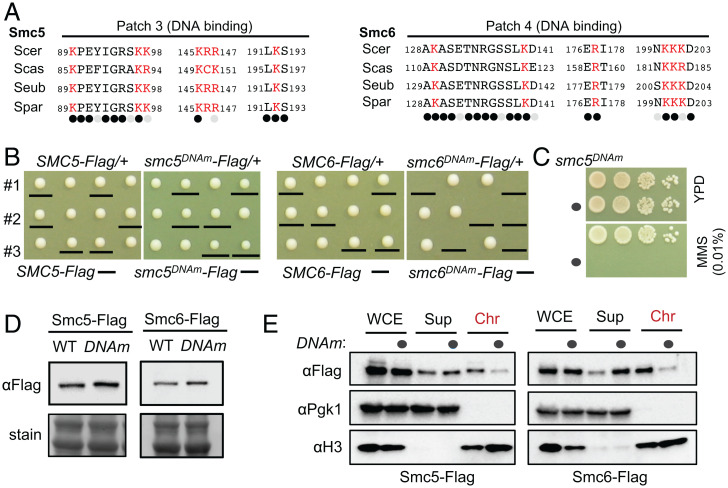
The effects of mutating the DNA binding sites on Smc5 and Smc6. (*A*) DNA binding sites on Smc5 (*Left*) and Smc6 (*Right*) contain mostly conserved residues. Labels are as in Fig. 4*A*. (*B*) The *smc5^DNAm^* allele leads to slow growth (*Left*), while the *smc6^DNAm^* allele leads to cell death (*Right*). (*C*) *smc5^DNAm^* leads to MMS sensitivity. (*D*) DNA binding site mutations in Smc5 and Smc6 do not affect their protein level. (*E*) The effects of DNA binding site mutations in Smc5 and Smc6 on their chromatin association.

*smc5^DNAm^* and *smc6^DNAm^* led to slow growth and cell death, respectively (Fig. 5 *A* and *B*). Similar to *nse4^DNAm^, smc5^DNAm^* cells showed hypersensitivity to MMS (Fig. 5*C*). While both *smc5^DNAm^* and *smc6^DNAm^* maintained their protein levels (Fig. 5*D*), they reduced the levels of chromatin associated proteins compared with wild-type controls (Fig. 5*E*). That *smc6^DNAm^* exhibited a more severe effect on growth than *smc5^DNAm^* is surprising and may suggest that it impairs a downstream step in DNA manipulation after loading onto chromatin, among other possibilities.

In summary, our data suggest that DNA binding sites of Smc5/6 subunits contribute to their chromatin association with differential effects on cell growth and genotoxin survival.

### CLMS analyses suggest multi-subunit conformational changes upon DNA engagement.

Recent CLMS and EM data suggest that DNA-free Smc5/6 has a closed arm configuration containing head-proximal Nse1-3 (10–14, 22). By contrast, the structure of DNA-bound Smc5/6 shows arm opening and Nse1-3 shifting to above the head regions, suggestive of major conformational changes. For a detailed understanding of these changes, we compared the available CLMS data derived from DNA-free Smc5/6 (10, 11) with the structure of DNA-bound Smc5/6. Mapping these CL pairs to our structure reveals specific changes in subunit associations and folding after DNA entrapment, as well as preserved contacts during the transformation. Cross-links that violate cross-linker distance restraints (25 to 30 Å), referred to as violated CLs, suggest conformational changes occurring upon DNA capture, whereas those satisfying the restraints, referred to as “satisfied CLs”, suggest relatively unchanged associations.

**Fig. 6.**
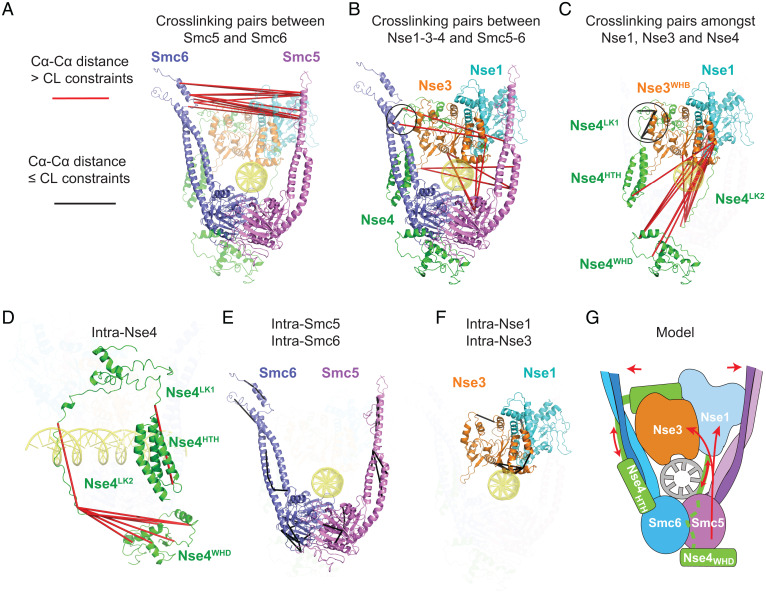
Mapping CLMS data derived from DNA-free Smc5/6 onto the structure of DNA-bound Smc5/6. (*A*) Interprotein CL pairs between Smc5 and Smc6 indicate arm opening. (*B*) Interprotein CL pairs connecting Nse1-3-4 with Smc5 or Smc6 suggest that Nse3 moves away from the Smc5 head/neck regions to be positioned above DNA. (*C*) Interprotein CL pairs between Nse1, Nse3 and Nse4 suggest that the Nse1-WHB and Nse3-WHA domain move away from the Nse4-WHD and adjacent linker 2 (Lk2) region. (*D*) Intra-Nse4 CL pairs are indicative of large conformational changes within Nse4 midregion. (*E*) Intra-subunit CL pairs for Smc5 and 6. (*F*) Intra-subunit CL pairs for Nse1 and Nse3. (*G*) A model of Smc5/6 conformational changes upon DNA binding. The arrows indicate conformational changes in Smc5/6 upon encircling of DNA.

We applied unified criteria to reanalyze CLMS datasets of DNA-free Smc5/6 from our group (10) and those from the Stephan Gruber group (11) and derived close to 100 CL pairs mappable to the current structure of the complex (*SI Appendix*, Table S2 and Methods). The two DNA-free Smc5/6 datasets gave similar and internally consistent conclusions. They reveal that the majority of violated CLs in DNA-bound Smc5/6 structure occur between subunits, while satisfied CLs are located mostly within the same subunit, with the exception of Nse4. These results suggest that while subunits shift position relative to each other upon DNA binding, individual subunit structures do not undergo major changes, with the exception of Nse4. Specifically, we identified four groups of changes upon DNA binding, as well as a pivot point that may serve as an anchor during transformation (Fig. 6, *SI Appendix*, Fig. S6 and Table S2).

First, all 14 cross-links connecting Smc5 and Smc6 arms in DNA-free complex violate cross-link distances when mapped to the DNA-bound Smc5/6 structure, with cross-linked residues now separated by up to 86 Å (Fig. 6*A* and *SI Appendix*, Table S2). This result confirms arm-opening upon DNA binding as mentioned above and provides a validation for our analysis.

Second, we found that Nse3 shifts position relative to Smc5 and 6 (Fig. 6*B*). Eight violated CLs are between residues of the Nse3-WHA or WHB domains and those of the SMC neck or head regions, with maximal pair distance of 70 Å in the DNA-bound Smc5/6 structure (Fig. 6*B*and *SI Appendix*, Table S2). We conclude that DNA engagement entails moving Nse3 from head/neck regions toward the direction of the hinge.

Third, Nse1 moves along with Nse3 upon DNA binding as shown by their similar cross-linking changes relative to Nse4 (Fig. 6*C* and *SI Appendix*, Fig. S6). A total of 13 cross-links connecting Nse1 or Nse3 with Nse4-WHD and its linker regions are violated CLs, with a maximal Cα-Cα pair distance of 113 Å in the current structure (Fig. 6*C*, *SI Appendix*, Fig. S6 and Table S2). Among these are three DNA-binding lysine residues in the Nse3-WHA domain (K50, K66, K122), suggesting that they move away from Nse4-WHD in order to engage with DNA (Fig. 6*C*, *SI Appendix*, Fig. S6 and Table S2). We further identified seven satisfied CLs connecting Nse4 around its Nse3-BD domain to either Nse3 or Smc6 neck region, indicating interactions retained during structural transformation (Fig. 6 *B* and *C*, circles, and *SI Appendix*, Table S2).

Fourth, all eight CLs involving the midsection of Nse4 are violated CLs in the DNA-bound Smc5/6 structure, suggesting regional extension and moving toward the hinge direction (Fig. 6*D*, and *SI Appendix*, Table S2). This is in striking contrast to CLs within Smc5, Smc6, Nse1 and Nse3, which are all satisfied CLs when mapped to our structure (Fig. 6 *E* and *F*), suggesting that these subunits, at least the parts seen in the current structure of the DNA-bound complex, sustained largely unaltered conformations during DNA capture.

In summary, our analyses suggest multiple conformational changes upon DNA encirclement, including arm opening, Nse1-3 translocation from the head/Nse4-WHD region toward the hinge direction, and the expansion and upward movement of the Nse4 midregion (Fig. 6*G*). A small area formed by parts of the Smc6 neck, Nse3, and the kleisin’s Nse3-BD may serve as a pivot point during transformation.

## Discussion

Our cryo-EM structure of DNA-bound Smc5/6 and accompanying CLMS analyses provide important insight into how Smc5/6 engages with DNA. We identify multi-subunit interactions, including the scaffolding role of hook shaped Nse4 kleisin, that support formation of a DNA clamp enclosing two turns of DNA, as well as subunit shifts to enable DNA clamping. We further pinpoint the multiple DNA binding sites involving Smc5-6 and Nse3-4 and their different effects on Smc5/6-chromatin association and cellular fitness. Our analyses further reveal both pan-SMC and Smc5/6-specific features in trapping DNA. Collectively, our findings provide insights into the Smc5/6-DNA complex structure and functional implications.

### A common SMC DNA-clamp mechanism with distinct complex-specific features.

Recent structural data reveal that cohesin and condensin form a DNA clamp encircling DNA within a tunnel formed by multiple subunits (3–7). This tunnel is located in the so-called E-K compartment between engaged SMC-head regions and kleisin. The structure of Smc5/6 reveals a similar DNA clamp architecture with DNA enclosed in the E-K compartment (Fig. 1 *B* and *C*). In all three cases, the E-head regions hold DNA from the bottom, while the top of the DNA is secured by a KITE subunit in Smc5/6 (*SI Appendix*, Fig. S7*A*) or HAWK subunits in cohesin (*SI Appendix*, Fig. S7*B*) and condensin (3–7). In this sense, HAWK and KITE subunits serve the same purpose. Thus, despite the differences in subunit composition, DNA clamping within the E-K compartment is a unified theme for all three eukaryotic SMC complexes.

However, it is also important to note key differences among these SMC complexes. For example, as mentioned above, while kleisins in cohesin and condensin employ their N-terminal HTH domain to bind DNA (3–7), Nse4 uses it midregion to nudge against DNA. In addition, cohesin and condensin entrap longer pieces of DNA than does Smc5/6 (3–7). Moreover, DNA bending is uniquely observed for cohesin (3–5). It is likely that these differences contribute to the divergent roles seen for the three eukaryotic SMC complexes.

DNA clamping in the E-K compartment was also shown for the prokaryotic MukBEF complex (*SI Appendix*, Fig. S7*C*) (17). In this complex, both copies of the dimeric MukE KITE subunits contact DNA (17). This is in contrast to Smc5/6 wherein only the Nse3 KITE contacts DNA. Differences are also seen in the manner of KITE-DNA binding sites. MukE uses a loop region, while Nse3 mainly employs two helices (17). When comparing the kleisin subunits, the MukF kleisin and Nse4 both form a hook-shaped structure linking all parts of the complex and DNA; however, the kleisin-DNA and kleisin-KITE interactions show differences (17). Another intriguing feature differentiating MukBEF from eukaryotic SMC complexes is its lack of arm tilting relative to the DNA (17). The significance of this difference is currently unclear but may reflect functional differences or distinct states of the complexes given that the MukBEF structure was captured during DNA unloading (17).

Overall, comparing the DNA-bound Smc5/6 structure with those of cohesin, condensin, and MukBEF, a fundamental shared feature of the SMCs appears to be DNA clamping in the E-K compartment (*SI Appendix*, Fig. S7). Detailed differences noted above regarding Smc5/6 may confer specificity of Smc5/6-mediated functions. Testing this hypothesis in the future will help further elucidate SMC-based DNA manipulation.

### Multi-subunit conformational changes enable Smc5/6 to encircle DNA.

We analyzed CLMS data derived from DNA-free Smc5/6 against the DNA-bound Smc5/6 structure and provided insights into conformational changes that enable Smc5/6 encirclement of DNA (Fig. 6). We found that intrasubunit CL pairs within Smc5, Smc6, Nse1 and Nse3 derived from the DNA-free complex satisfy cross-link distance constraints in the DNA-bound structure, suggesting that the structural folds of these subunits are largely maintained during the transition between the two states. However, the opposite was found for the Nse4 linker regions; our analyses suggest that these flexible regions stretch and move from the head-proximal region toward the direction of the hinge region. The Nse4 transformation is consistent with the conclusions that Nse1-3 also shifts in the direction of the hinge, accompanied by arm opening. These changes are in contrast with interactions that may provide a local anchorage during the complex transformation.

We note that MukBEF’s KITE subunits shift in a manner resembling Nse1-3 and that arm opening is common among SMC complexes upon DNA clamping. They represent changes shared among SMC complexes (3–7, 17). It will be interesting in the future to test whether other SMC complexes also show a kleisin midregion expansion and movement or possess a pivot point as revealed here for Smc5/6.

### Differential effects of DNA binding sites from four subunits of Smc5/6.

Functional examination of DNA binding sites in Smc5 and 6 and Nse3 and 4 found that mutating each set of DNA binding residues reduced Smc5/6 chromatin association and cellular fitness, however the extent of the effects differed (Figs. 4 and 5). This could reflect different levels of reduction in DNA binding affinity among these mutants or differential effects on downstream DNA transaction steps, among other possibilities. We found that mutating Nse3 and Smc5-6 DNA binding sites exerted stronger effects on chromatin association than those of Nse4. As only Nse3 and Smc6 sites are required for viability, these sites might have additional roles in supporting Smc5/6 functions, a topic for future exploration. Perturbing Smc5- or Nse4-DNA binding mainly led to strong DNA damage sensitivity, suggesting that optimal Smc5/6-DNA association could be particularly important for genotoxin survival. Though Nse1 does not contact DNA in the current structure, it interacts extensively with Nse3-4, and thus may play a regulatory role in Nse3-4 association with DNA. We note that the RING domain of Nse1 that mediates its ubiquitin E3 function (20, 23, 24) appears to be less accessible upon DNA binding, which may suggest DNA-mediated regulation of E3 activity.

### Summary.

In conclusion, our integrative study has revealed the structure of DNA-bound Smc5/6 complex, identified the specific conformational changes required for DNA entrapment, and uncovered differential effects of DNA binding sites among four of its subunits. These findings help to define common SMC features and unique Smc5/6 attributes, as well as suggest potential functional implications. They also provide an important foundation for further investigation into the genome protection functions of Smc5/6 against both endogenous and exogenous genome stressors.

## Materials and Methods

### Purification of *S. cerevisiae* hexameric Smc5/6 complex.

The complex composed of Smc5 (E1015Q), Smc6 (E1048Q), and Nse1-4 was purified as described (11). Briefly, complex-bearing plasmid received from Stephan Gruber was expressed in BL21 (DE3) Rossetta cells with standard induction conditions. Cells were lysed in lysis buffer (50 mM Tris⋅HCl, pH 7.5, 300 mM NaCl, 5% glycerol, 25 mM imidazole) in the presence of 2 mM DTT, 1 mM phenylmethylsulfonyl fluoride and benzonase (750 U/100 mL). After removing debris by centrifugation at 40,000*g* for 1 h, supernatant was loaded onto Strep column (5 mL) to pull down the complex tagged with 3C-Twin-Strep. After washing the column with 50 mL lysis buffer, Smc5/6 was eluted with lysis buffer supplemented with 2.5 mM desthiobiotin and loaded on Heparin column (1 mL). After washing the column with 10 mL lysis buffer, Smc5/6 was eluted with 50 mM Tris⋅HCl, pH 7.5, 800 mM NaCl, 5% glycerol. The elution was concentrated and applied to a superpose 6 increase column equilibrated with 25 mM Hepes, pH 7.5, 250 mM NaCl, 1 mM DTT to obtain peak fractions containing the complex (*SI Appendix*, Fig. S1).

### Cryo-EM analyses of ATP and dsDNA-bound Smc5/6.

Smc5/6 (0.3 mg/mL) was incubated with 1 mM ATP, 2 mM MgCl_2_ and 1.5 μM dsDNA (5′-TGGTTTTTATATGTTTTGTTATGTATTGTTTATTTTCCCTTTAATTTTAGGATATGAAAACAAGAATTTATC-3′ and its complementary strand) at 4 °C for 2 h. This sample was applied onto glow-discharged UltrAuFoil 300 mesh R1.2/1.3 grid. Grids were blotted for 2.0 s at 4 °C, 100% humidity, and flash frozen in liquid ethane using a FEI Vitrobot Mark IV. All images were collected on a FEI Titan Krios electron microscope operated at an acceleration voltage of 300 kV with a Gatan K3 camera with a 1.069 Å pixel size. Movies were recorded in counting mode at an electron dose rate of 30 e−/pixel/s with a total exposure time of 2 s and frames rate 50 ms per /frame, for an accumulated electron dose of 53.55 e−/Å^2^. Motion correction was performed with MotionCor2 (25), and contrast transfer function parameters were estimated by Ctffind4 (26). All other steps of image processing were performed by RELION 3.0 (27) and Cryosparc v3.3.0 (28). After blob picking and crYOLO (29) picking from 9,286 images and multirounds of 3D classification, a total of 240,130 particles were selected for local 3D classification of Nse4 WHD in RELION 3.0. Two of the 3D classes with good secondary structural features and the corresponding 201,249 particles were polished using RELION, yielding an electron microscopy map with a resolution of 3.8 Å after 3D auto-refinement and postprocessing. All reported map resolutions are from gold-standard refinement procedures with the Fourier shell correlation cutoff being 0.143 criterion after postprocessing by applying a soft mask. Model-building of ATP-dsDNA-SMC5/6 atomic structure was performed manually based on the cryo-EM density map and computed model (30) by using COOT4 (31). The model was then refined against the cryo-EM density map using phenix.real space_refine by applying geometric and secondary structure restraints (32). All figures were prepared by PyMol (https://pymol.org/2/) or UCSF Chimera (33). Details of data collection, image processing and model building are shown in *SI Appendix*, Table S1 and Fig. S2.

### CLMS data analysis.

CLMS data used for analysis was taken from Yu et al. (2021) (10) and from reanalysis of data from Taschner et al. (11). MS raw files from the latter were downloaded from the PRIDE database (PXD02416) and searched using similar parameters as reported (10) to provide a common basis of comparison between the two datasets. CLMS search was performed with XlinkX in Proteome Discoverer v2.2 against database containing Smc5-6 and Nse1-6 with following parameters: Cross-linker PhoX (K,S,T,Y) +209.9782. Static modifications for carbamidomethylation (C) +57.021. Dynamic modifications for oxidation (M) +15.995, PhoX hydrolyzed +227.982 (K,S,T,Y), PhoX Tris-hydrolyzed +331.046 (K,S,T,Y). Trypsin was set as enzyme with up to 3 maximum missed cleavages. Precursor mass tolerance of 10 ppm, FTMS Fragment Mass Tolerance of 20 ppm and ITMS Fragment Mass Tolerance of 0.5 Da. Percolator FDR rate was set to 1%. Matches with XlinkX scores less than 40 were removed. Remaining spectra were manually inspected to remove ambiguous assignments with insufficient b- or y- ions to specifically assign peptide backbone sequence. Cross-links and cross-link distances were mapped and measured using PyXlinkViewer (34) in Pymol v2.5.2. Cross-link distance constraint of 30 Å and 25 Å were used for cross-linkers disuccinimidyl sulfoxide and PhoX respectively. Cross-links mapped with distances larger than or equal to the distance constraint are considered to have violated the constraint while cross-links mapped with distances smaller than the constraint are considered to have satisfied the constraint. We note that both datasets are derived from the eight subunit Smc5/6 complex, whose CLMS data are highly similar to those of the hexamer analyzed here (11), thus providing a good basis of comparison. Our results of these wild-type Smc5/6 complexes in the presence or absence of γATP are agreeable as well.

### Yeast strains and mutant analyses.

All yeast strains are in W303 background containing wild-type *RAD5*. To generate DNA binding site mutations for each gene studied, DNA fragment containing the mutations was synthesized and then fused with a tag containing Flag and a selection marker using PCR-based method. The resultant PCR product was used to transform diploid yeast cells. The transformants were screened for the correct gene replacement of one of the two alleles by PCR-based diagnosis method. The positive clones were further analyzed by sequencing the entire targeted gene locus to identify those only containing the desirable mutations and no other mutation. Such diploids were sporulated and examined by tetrad analyses that involved at least 12 tetrads. In all cases, two independent biologically isolated diploids were examined, and they gave the same results. In the case of Nse4 and Smc5 DNA binding mutants, haploid cells were spotted on plates in the presence or absence of MMS to assess genotoxic sensitivity. In all cases, yeast cells were grown at 30 °C. The controls with tagged wild-type genes were examined in the same manner as the cells containing the mutated genes.

### Protein examination and chromatin fractionation.

Diploid cells containing one copy of the DNA binding mutant tagged with a Flag module and one copy of untagged wild-type gene for Nse3, Smc5 and Smc6 were examined for protein levels using TCA (trichloroacetic acid) method as described (35). In brief, log-phase growing cells were lysed by bead beating in the presence of 20% trichloroacetic acid. The pellets were recovered by centrifugation and incubated with 1× Laemmli buffer at 95 °C for 5 min to recover proteins. Subsequently, proteins were separated on 4 to 20% Mini-PROTEAN TGX gels (Bio Rad) followed by Western blotting with anti-Flag antibody (Sigma). Equal loading was assessed by staining the membrane with Ponceau S.

Chromatin fractionation was performed as described in diploid cells for Nse3, Smc5 and Smc6 (36). Briefly, spheroplasts from log-phase cells were lysed using extraction buffer (20 mM pH 6.6 Pipes-KOH, 150 mM KOAc, 2 mM Mg(OAc)2, 1 mM NaF, 0.5 mM Na_3_VO_4_, 1× Sigma protease inhibitors, 1% Triton X-100) for 5 min on ice. Lysates were centrifuged at 16,000 × g for 15 min on a sucrose cushion. Chromatin pellets were washed and resuspended with extraction buffer. Protein loading buffer was added to all fractions and boiled for 5 min followed by sodium dodecyl sulfate-polyacrylamide gel electrophoresis (SDS-PAGE) and Western blotting. Flag-tagged proteins were detected by anti-Flag antibody (Sigma). Histone H3 was used as the marker for chromatin-associated proteins and was detected by an anti-H3 antibody (ab1791, Abcam). Pgk1 was used as a marker for nonchromatin-associated proteins and was detected by an anti-Pgk1 antibody (22C5D8, Invitrogen). For Nse4, haploid cells containing either the Flag tagged wild-type of mutant proteins were examined by TCA method for protein levels and by chromatin fractionation.

## Supplementary Material

Supplementary File

## Data Availability

The atomic coordinates of the ATP- and dsDNA-bound 6-subunit Smc5/6-E/Q complex have been deposited in the Research Collaboratory for Structural Bioinformatics Protein Data Bank with the code 7TVE. Cryo-EM density maps have been deposited in the Electron Microscopy Data bank with accession code EMD-26140. All study data are included in the article and/or supporting information.
