# Supplementary material for: Cryo-EM structure of DNA-bound Smc5/6 reveals DNA clamping enabled by multi-subunit conformational changes
Source: Proc Natl Acad Sci U S A. 2022 Jun 1;119(23):e2202799119. doi: 10.1073/pnas.2202799119 (PMC9191643; doi:10.1073/pnas.2202799119)
Supplement: Supplementary File [file pnas.2202799119.sapp.pdf]

Supplementary Material for

Cryo-EM structure of DNA-bound Smc5/6 reveals DNA clamping enabled by multi-subunit conformational changes

You Yu[1#], Shibai Li[2#], Zheng Ser[3],
Huihui Kuang[4,] Thane Than[2], Danying Guan[2],
Xiaolan Zhao[2]* and Dinshaw J. Patel[1]*

[1] Structural Biology Program, Memorial Sloan-Kettering Cancer Center, New York, NY, 10065, USA

[2] Molecular Biology Program, Memorial Sloan-Kettering Cancer Center, New York, NY, 10065, USA

[3] Functional Proteomics Laboratory, Institute of Molecular and Cell Biology, Agency for Science, Technology and Research (A*STAR), Singapore 138673, Singapore

[4] Simons Electron Microscopy Center, New York Structural Biology Center, New York, NY, 10027, USA

# co-first authors

[*] Corresponding authors: zhaox1@mskcc.org (X.Z.) and pateld@mskcc.org (D.J.P.)

Supplementary Figure Legends

Fig. S1. Sample preparation of 6-subunit *S. cerevisiae* Smc5/6-E/Q complex. Sizing column (Superose 6 increase) elution profile and Coomassie stained gel picture showing the Smc5/6 subunits in the peak fraction and the EM samples containing the complex, dsDNA, and ATP.

Fig. S2. Cryo-EM reconstruction of DNA-bound Smc5/6 complex. (*A*) Workflow of cryo-EM image processing for the complex. (*B*) Global Fourier Shell Correlation (FSC) curve of the complex. The curve for the two half datasets is in blue and that for the refined model versus the cryo-EM map is in red. The overall cryo-EM map resolution is 3.8 Å with FSC set at 0.143. (*C*) Angular distribution plot of final 3D EM map for the complex. (*D*) Final 3D reconstructed map of the complex colored according to local resolution.

Fig. S3. Side chain identification in the cryo-EM structure of DNA-bound Smc5/6 and structural details. (*A*) A view of the complex in ribbon representation with density map shown as a transparent surface. Inserts outline examples where amino acid side chains can be fitted in the density map. (*B, C*) An electron density representation (B) and a ribbon representation (C) of the DNA-bound Smc5/6 structure. These views are rotated 180º compared with those in Figure 1B and 1C. (*D*) “Engaged” Smc1 and Smc3 head domains in the structure of cohesion in the ATP- and DNA-bound Smc1/3-E/Q complex (PDB: 6zz6)[1]. (*E*) ”Juxtaposed” Smc2 and Smc4 head domains in the structure of apo-condensin (PDB: 6YVU)[2].

Fig. S4. Sequence alignment and comparison of Nse3 proteins from *X. laevis* and *S. cerevisiae*. (*A*) Sequence alignments between the Nse3 domains of *S. cerevisiae* and *X. laevis.* Residues involved in hydrogen bond interactions with DNA are marked by stars for the Nse3-patch 1 region (blue) and the Nse3-mediated patch 2 region (green). The linker between the WHA and WHB of Nse3 in yeast contains insertions including a helix (adH) and a loop (WHL). (*B*) Structural overlay between the Nse1-3 and the interacting Nse4 peptide from *X. laevis* (grey) and *S. cerevisiae* (color).

Fig. S5. DNA binding details of the DNA-bound Smc5/6 complex. (*A, B*) Overview of DNA contacts involving Nse3 (patches 1 and 2) and Nse4 (patch 5) (A) and those involving Smc5 (patch 3) and Smc6 (patch 4) (B). Observable Lys and Arg side chains are shown in a ribbon representation, while those whose side chain densities could not be traced are shown in a ball representation.

Fig. S6. CLMS data from DNA-free Smc5/6 mapped to the DNA-bound Smc5/6 structure. Inter-subunit CLs detected among Nse1, Nse3 and Nse4. Red lines indicate CL pairs with Cα-Cα distance exceeding that allowed by crosslinkers, while black lines indicate satisfied CL pairs.

Fig. S7. SMC-DNA clamping in the E-K compartment. The protein subunits are shown in a ribbon representation and the bound dsDNA in a yellow stick representation. (*A*) 3.8 Å cryo-EM structure of dsDNA-bound *S. cerevisiae* Smc5/6-EQ complex (this study). (*B*) 3.4 Å cryo-EM

structure of dsDNA-bound *S. cerevisiae* cohesion-EQ complex (PDB: 6ZZ6)[1]. (*C*) 3.1 Å cryo-EM structure of dsDNA-bound *E. coli* MukBEF-EQ complex (PDB: 7NYW)[3].

References

1. Collier, J. et al. Transport of DNA within cohesin involves clamping on top of engaged heads by Scc2 and entrapment within the ring by Scc3. *elife* **9**, e59560 (2020).

2. Lee, B.-G. et al. Cryo-EM structures of holo condensin reveal a subunit flip-flop mechanism. *Nat Struct Mol Biol* **27**, 743-751 (2020).

3. Burmann, F., Funke, L. F. H., Chin, J. W. & Lowe, J. Cryo-EM structure of MukBEF reveals DNA loop entrapment at chromosomal unloading sites. *Mol Cell* **81**, 4891-4906 (2021).

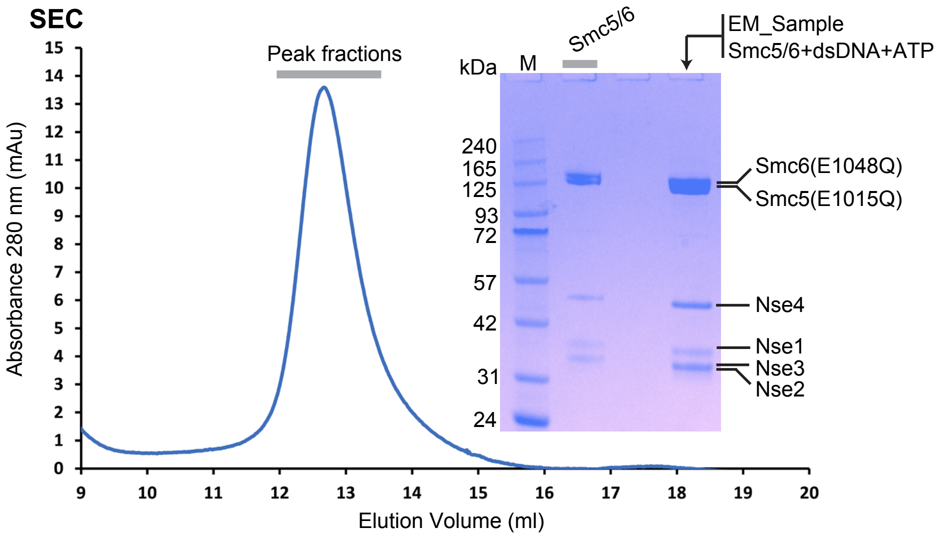
SEC
Peak fractions
Absorbance 280 nm (mAu)
Elution Volume (ml)
15
14
13
12
11
10
9
8
7
6
5
4
3
2
1
0
9
10
11
12
13
14
15
16
17
18
19
20
kDa
M
Smc5/6
EM_Sample
Smc5/6+dsDNA+ATP
240
165
125
93
72
57
42
31
24
Smc6(E1048Q)
Smc5(E1015Q)
Nse4
Nse1
Nse3
Nse2

Figure S1

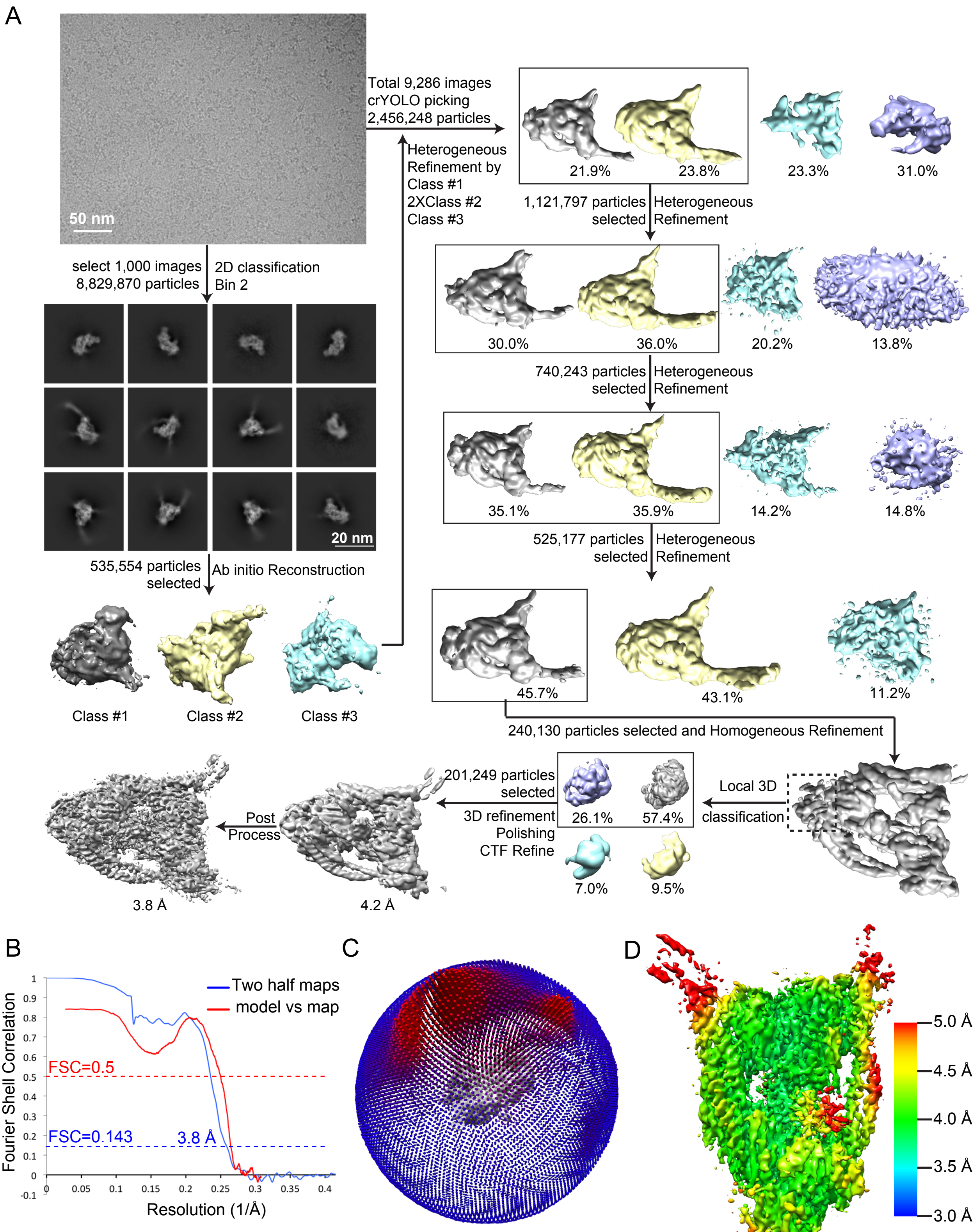
A
50 nm
Total 9,286 images
crYOLO picking
2,456,248 particles
Heterogeneous
Refinement by
Class #1
2XClass #2
Class #3
21.9%
23.8%
23.3%
31.0%
1,121,797 particles selected
Heterogeneous Refinement
select 1,000 images
8,829,870 particles
2D classification
Bin 2
30.0%
36.0%
20.2%
13.8%
740,243 particles selected
Heterogeneous Refinement
35.1%
35.9%
14.2%
14.8%
20 nm
535,554 particles selected
Ab initio Reconstruction
525,177 particles selected
Heterogeneous Refinement
45.7%
43.1%
11.2%
Class #1
Class #2
Class #3
240,130 particles selected and Homogeneous Refinement
201,249 particles selected
3D refinement
Polishing
CTF Refine
26.1%
57.4%
7.0%
9.5%
Local 3D classification
Post Process
3.8 Å
4.2 Å
B
Fourier Shell Correlation
Two half maps
model vs map
FSC=0.5
FSC=0.143
3.8 Å
Resolution (1/Å)
C
D
5.0 Å
4.5 Å
4.0 Å
3.5 Å
3.0 Å

Figure S2

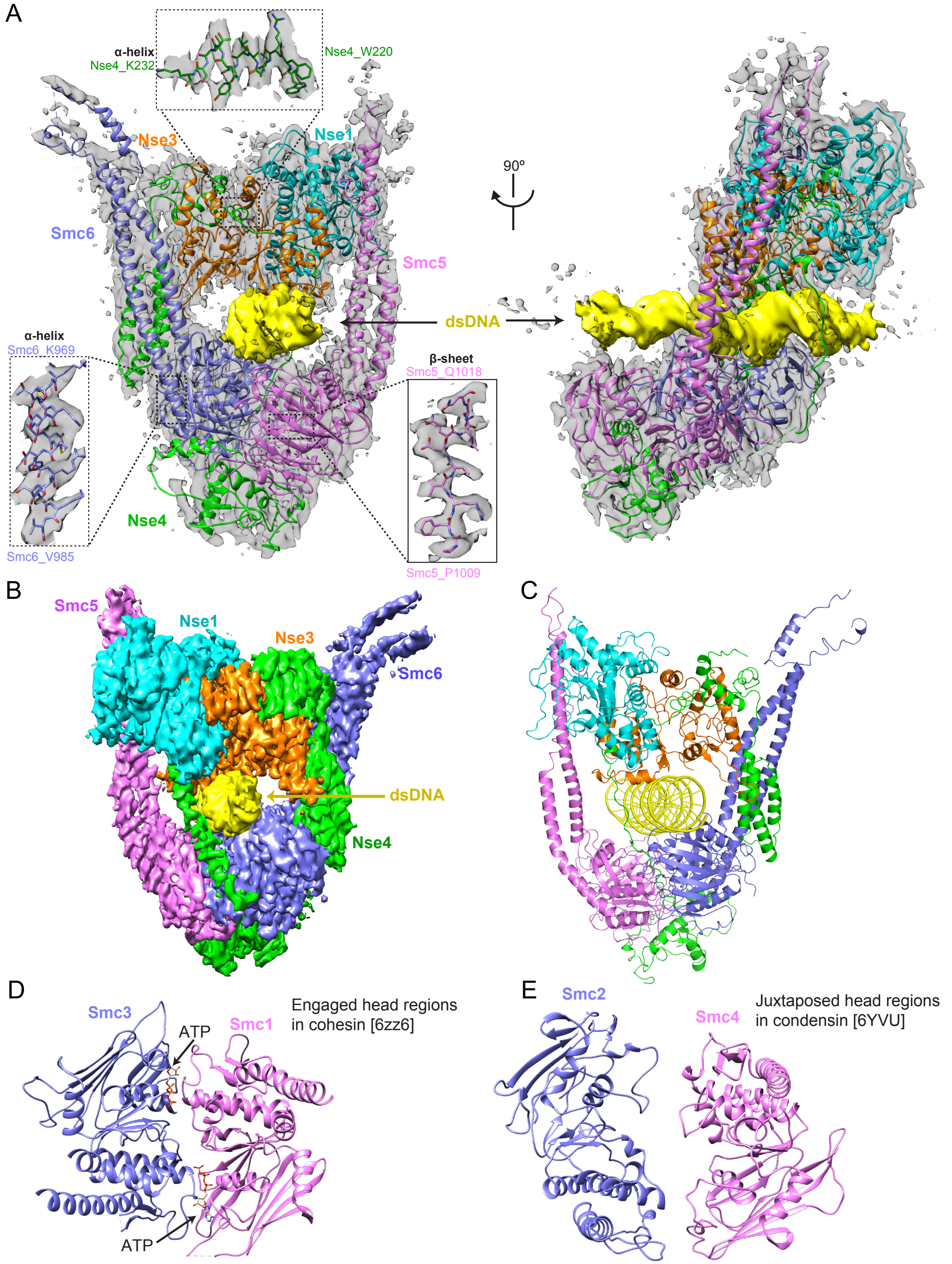
A
α-helix
Nse4_K232
Nse4_W220
Nse3
Nse1
Smc6
Smc5
90º
dsDNA
α-helix
Smc6_K969
β-sheet
Smc5_Q1018
Nse4
Smc6_V985
Smc5_P1009
B
Smc5
Nse1
Nse3
Smc6
dsDNA
Nse4
C
D
Smc3
ATP
Smc1
Engaged head regions
in cohesin [6zz6]
ATP
E
Smc2
Smc4
Juxtaposed head regions
in condensin [6YVU]

Figure S3

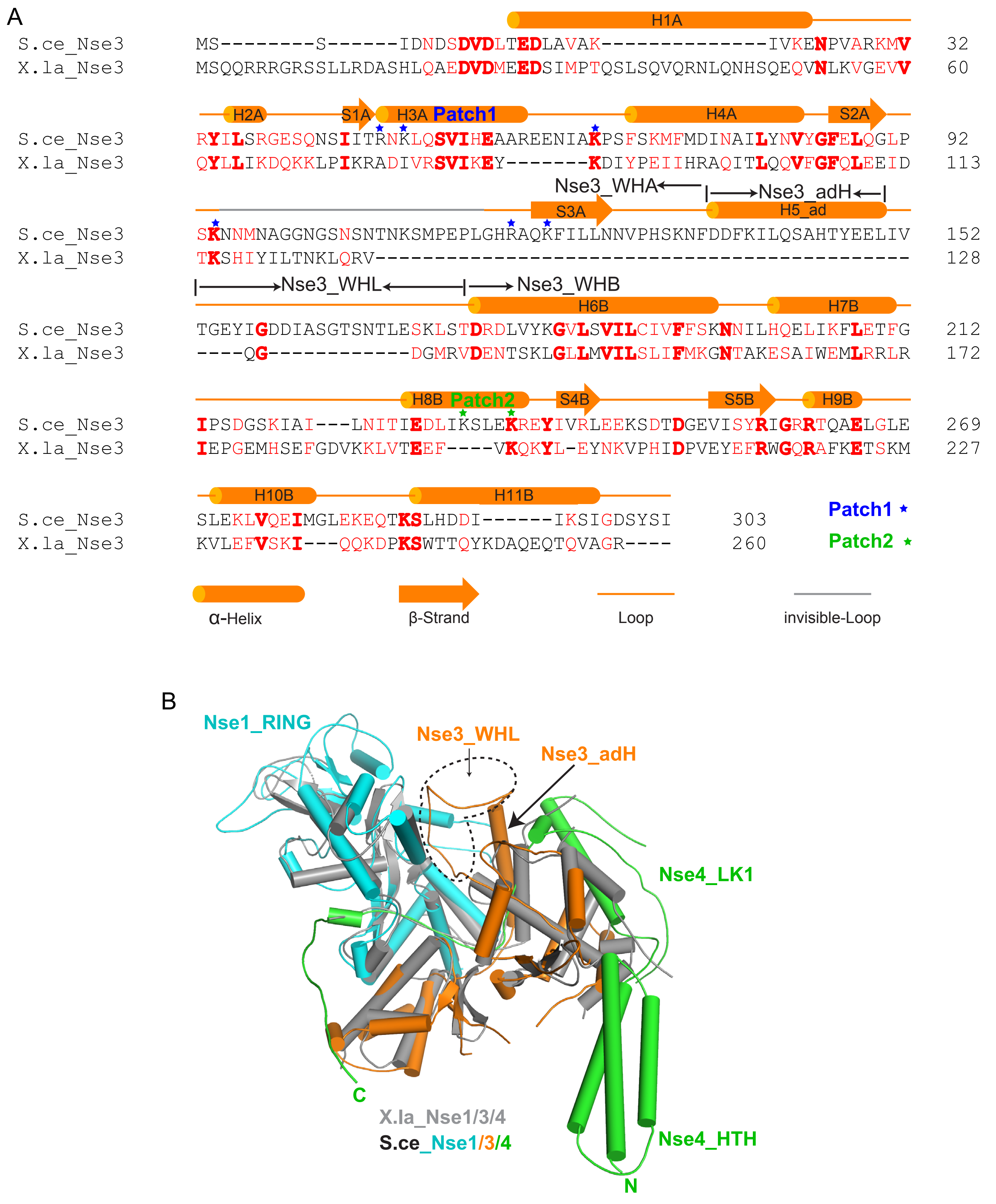
A
H1A
S.ce_Nse3 MS--------S------IDNDSDVDLTEDLAVAK--------------IVKENPVARKMV 32
X.la_Nse3 MSQQRRRGRSSLLRDASHLQAEDVDMEEDSIMPTQSLSQVQRNLQNHSQEQVNLKVGEVV 60
H2A S1A H3A Patch1 H4A S2A
S.ce_Nse3 RYILSRGESQNSIITRNKLQSVIHEAAREENIAKPSFSKMFMDINAILYNVYGFELQGLP 92
X.la_Nse3 QYLLIKDQKKLPIKRADIVRSVIKEY-------KDIYPEIIHRAQITLQQVFGFQLEEID 113
Nse3_WHA
Nse3_adH
S3A
H5_ad
S.ce_Nse3 SKNNMNAGGNGSNSNTNKSMPEPLGHRAQKFILLNNVPHSKNFDDFKILQSAHTYEELIV 152
X.la_Nse3 TKSHIYILTNKLQRV--- 128
Nse3_WHL
Nse3_WHB
H6B H7B
S.ce_Nse3 TGEYIGDDIASGTSNTLESKLSTDRDLVYKGVLSVILCIVFFSKNNILHQELIKFLETFG 212
X.la_Nse3 ----QG------------DGMRVDENTSKLGLLMVILSLIFMKGNTAKESAIWEMLRRLR 172
H8B Patch2 S4B S5B H9B
S.ce_Nse3 IPSDGSKIAI---LNITIEDLIKSLEKREYIVRLEEKSDTDGEVISYRIGRRTQAELGLE 269
X.la_Nse3 IEPGEMHSEFGDVKKLVTEEF----VKQKYL-EYNKVPHIDPVEYEFRWGQRAFKETSKM 227
H10B H11B
S.ce_Nse3 SLEKLVQEIMGLEKEQTKSLHDDI------IKSIGDSYSI 303
X.la_Nse3 KVLEFVSKI---QQKDPKSWTTQYKDAQEQTQVAGR---- 260
Patch1 ★
Patch2 ★
α-Helix
β-Strand
Loop
invisible-Loop
B
Nse1_RING
Nse3_WHL
Nse3_adH
Nse4_LK1
C
X.la_Nse1/3/4
S.ce_Nse1/3/4
Nse4_HTH
N

Figure S4

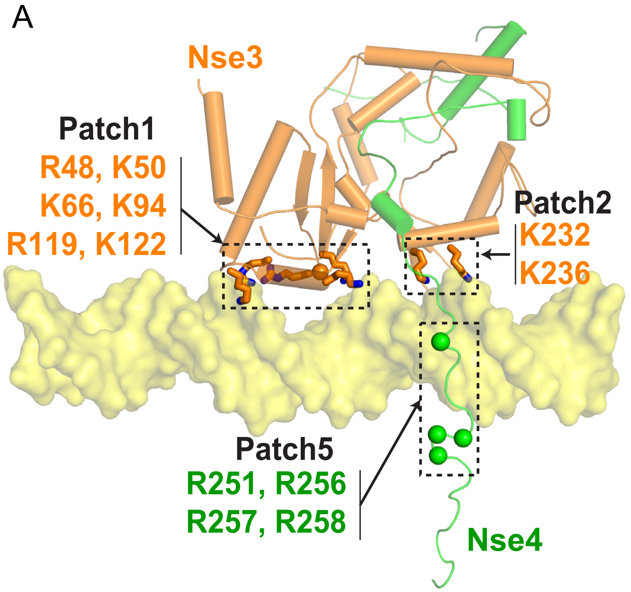
A
Nse3
Patch1
R48, K50
K66, K94
R119, K122
Patch2
K232
K236
Patch5
R251, R256
R257, R258
Nse4

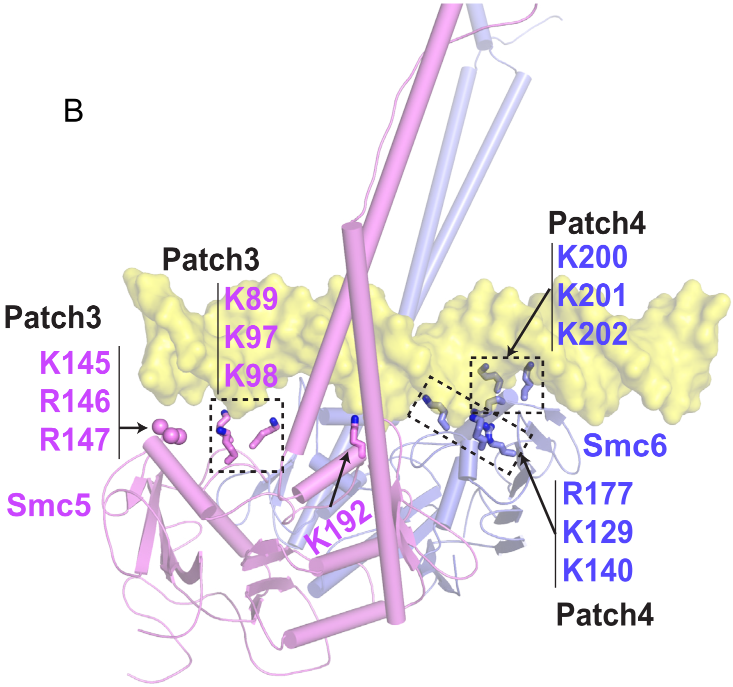
B
Patch3
K89
K97
K98
Patch3
K145
R146
R147
Smc5
K192
Patch4
K200
K201
K202
Smc6
R177
K129
K140
Patch4

Figure S5

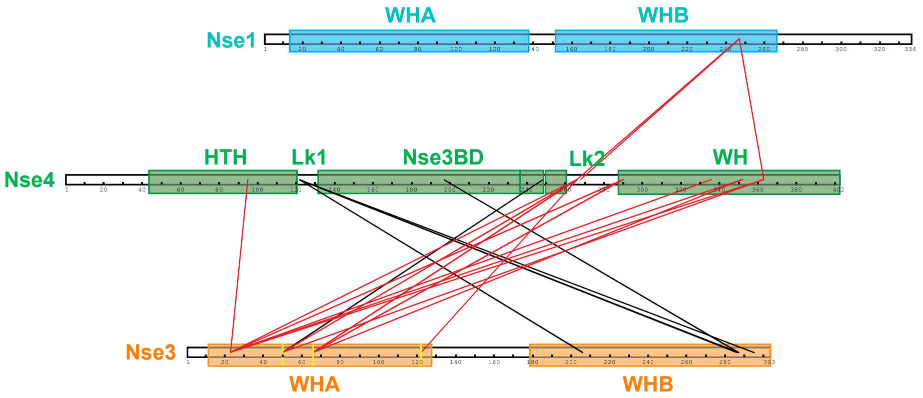

Figure S6

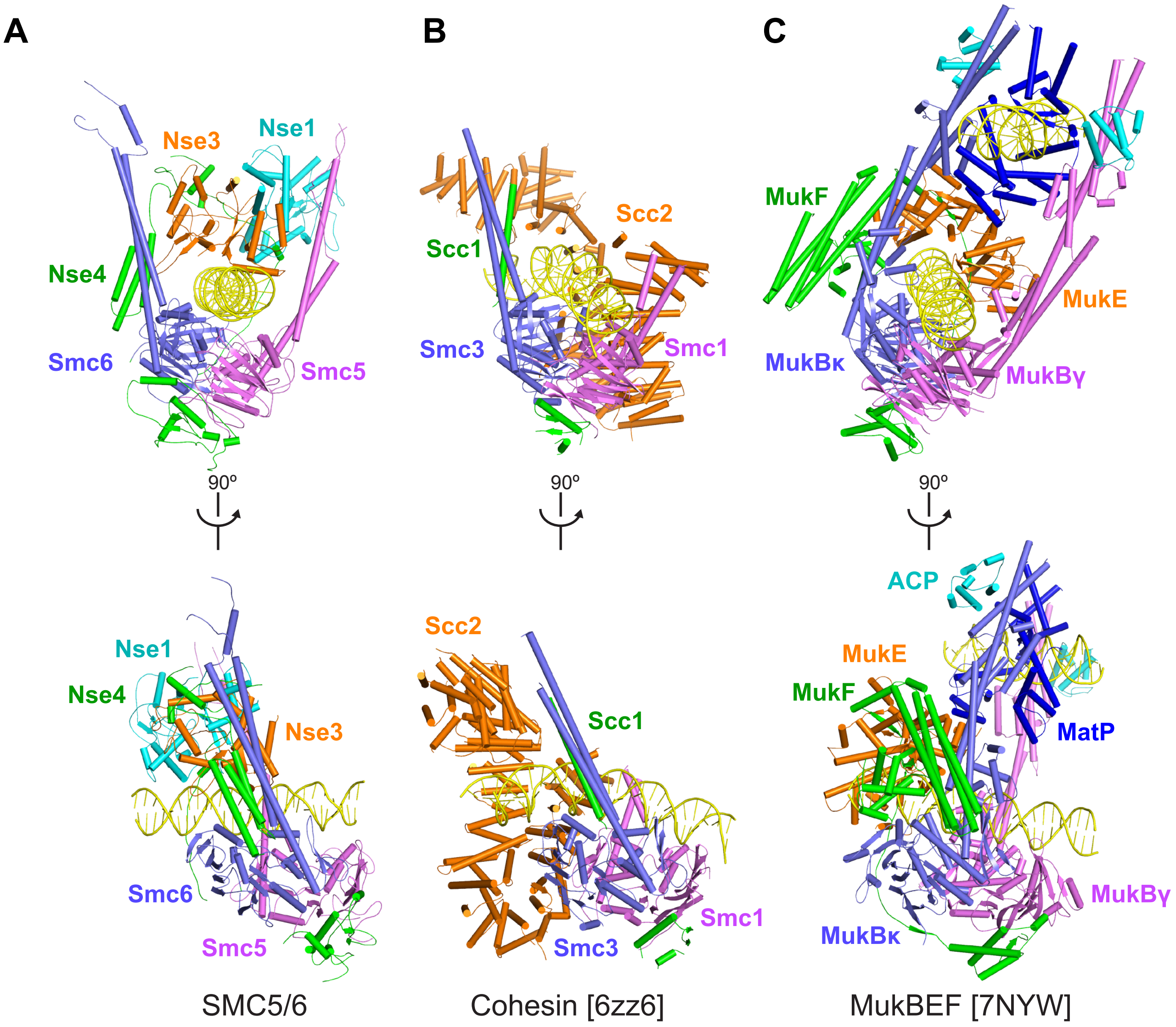
A
Nse3
Nse1
Nse4
Smc6
Smc5
90º
Nse1
Nse4
Nse3
Smc6
Smc5
SMC5/6
B
Scc2
Scc1
Smc3
Smc1
90º
Scc2
Scc1
Smc1
Smc3
Cohesin [6zz6]
C
MukF
MukE
MukBκ
MukBγ
90º
ACP
MukE
MukF
MatP
MukBγ
MukBκ
MukBEF [7NYW]

Figure S7

Table S1. Cryo-EM data collection, processing, and validation statistics

Sample	ATP-dsDNA-SMC5/6<br>EMD-26140<br>PDB: 7TVE
**Data collection**	
Microscope	Titan Krios
Detector	Gatan K3
Automation software	Leginon
Nominal magnification	81,000
Voltage (kV)	300 kV
Total dose (e^-/Å^2)	53.55
Dose rate (e^-/pixel/s)	30
Number of frames collected	40
Defocus range (µm)	-0.8 to -2.5
Pixel size (Å)	1.069
Collected Micrographs	9,286
Selected Micrographs	9,286
**Reconstruction**	
Initially autopicked particles	2,456,248
Particles used for classification	2,456,248
Particles in the final map	201,249
Symmetry	C1
Resolution	
FSC 0.5 (unmasked/masked, Å)	4.1/4.1
FSC 0.143 (unmasked/masked, Å)	3.8/3.8
Map sharpening B factor (Å^2)	-138.59
**Model composition**	
Protein residues	1793
Nonhydrogen atoms	15466
**Validation**	
MolProbity	2.78
Clash score	17.8
Map Correlation Coefficient	0.69
R.m.s. deviations	
Bond lengths (Å)	0.005
Bond angles (°)	0.827
Ramachandran plots	
Favored (%)	86.28
Allowed (%)	13.44
Outliers (%)	0.28
Rotamer outliers (%)	3.26

Table S2 CLMS analyses results used in this study

Protein 1	Residue 1	Protein 2	Residue 2	CA distance	Threshold	Sat-Viol	inter vs intra	figure panel	dataset
SMC5	248	SMC6	916	78.7	30	V	inter	Figure 6A	Yu et al 2021
SMC5	260	SMC6	906	85.6	30	V	inter	Figure 6A	Yu et al 2021
SMC5	248	SMC6	913	81.5	30	V	inter	Figure 6A	Yu et al 2021
SMC5	260	SMC6	913	78.6	30	V	inter	Figure 6A	Yu et al 2021
SMC5	259	SMC6	913	78.8	30	V	inter	Figure 6A	Yu et al 2021
SMC5	259	SMC6	906	86	30	V	inter	Figure 6A	Yu et al 2021
SMC5	259	SMC6	917	75.9	30	V	inter	Figure 6A	Yu et al 2021
SMC5	248	SMC6	917	77.5	30	V	inter	Figure 6A	Yu et al 2021
SMC5	252	SMC6	913	79	30	V	inter	Figure 6A	Yu et al 2021
SMC5	259	SMC6	913	78.8	25	V	inter	Figure 6A	Taschner et al 2021
SMC5	248	SMC6	913	81.5	25	V	inter	Figure 6A	Taschner et al 2021
SMC5	248	SMC6	921	82.9	25	V	inter	Figure 6A	Taschner et al 2021
SMC5	252	SMC6	913	79	25	V	inter	Figure 6A	Taschner et al 2021
SMC5	249	SMC6	913	79	25	V	inter	Figure 6A	Taschner et al 2021
NSE3	246	SMC5	909	73	30	V	inter	Figure 6B	Yu et al 2021
NSE3	236	SMC5	919	57.2	25	V	inter	Figure 6B	Taschner et al 2021
NSE3	236	SMC5	980	46.7	25	V	inter	Figure 6B	Taschner et al 2021
NSE3	236	SMC5	979	47.4	25	V	inter	Figure 6B	Taschner et al 2021
NSE3	246	SMC5	919	70.6	25	V	inter	Figure 6B	Taschner et al 2021
NSE3	23	SMC5	980	48.9	25	V	inter	Figure 6B	Taschner et al 2021
NSE3	66	SMC5	980	35.8	25	V	inter	Figure 6B	Taschner et al 2021
NSE3	23	SMC6	281	65.7	25	V	inter	Figure 6B	Taschner et al 2021
NSE4	197	SMC5	235	72.4	25	V	inter	Figure 6B	Taschner et al 2021
NSE4	197	SMC6	281	13.8	25	S	inter	Figure 6B	Taschner et al 2021
NSE1	247	NSE4	266	71	30	V	inter	Figure 6C	Yu et al 2021
NSE1	247	NSE4	268	75.3	30	V	inter	Figure 6C	Yu et al 2021
NSE1	247	NSE4	363	113.8	30	V	inter	Figure 6C	Yu et al 2021
NSE3	23	NSE4	363	85.3	30	V	inter	Figure 6C	Yu et al 2021
NSE3	206	NSE4	122	19.2	30	S	inter	Figure 6C	Yu et al 2021
NSE3	295	NSE4	122	10.3	30	S	inter	Figure 6C	Yu et al 2021
NSE3	23	NSE4	95	70	25	V	inter	Figure 6C	Taschner et al 2021
NSE3	23	NSE4	266	68.4	25	V	inter	Figure 6C	Taschner et al 2021
NSE3	23	NSE4	336	84.5	25	V	inter	Figure 6C	Taschner et al 2021
NSE3	23	NSE4	352	89	25	V	inter	Figure 6C	Taschner et al 2021
NSE3	50	NSE4	266	47	25	V	inter	Figure 6C	Taschner et al 2021
NSE3	50	NSE4	363	72.3	25	V	inter	Figure 6C	Taschner et al 2021
NSE3	**66**	NSE4	266	56.1	25	V	inter	Figure 6C	Taschner et al 2021
NSE3	**66**	NSE4	290	71.1	25	V	inter	Figure 6C	Taschner et al 2021
NSE3	**66**	NSE4	352	75.6	25	V	inter	Figure 6C	Taschner et al 2021
NSE3	**122**	NSE4	266	51.7	25	V	inter	Figure 6C	Taschner et al 2021
NSE3	50	NSE4	248	24.1	25	S	inter	Figure 6C	Taschner et al 2021
NSE3	286	NSE4	122	22.7	25	S	inter	Figure 6C	Taschner et al 2021
NSE3	287	NSE4	122	23	25	S	inter	Figure 6C	Taschner et al 2021
NSE3	287	NSE4	197	15.2	25	S	inter	Figure 6C	Taschner et al 2021
NSE4	95	NSE4	122	43.3	25	V	intra	Figure 6D	Taschner et al 2021
NSE4	248	NSE4	266	47.2	25	V	intra	Figure 6D	Taschner et al 2021
NSE4	266	NSE4	285	40.6	25	V	intra	Figure 6D	Taschner et al 2021
NSE4	266	NSE4	290	49.1	25	V	intra	Figure 6D	Taschner et al 2021
NSE4	266	NSE4	352	62.6	25	V	intra	Figure 6D	Taschner et al 2021
NSE4	266	NSE4	363	65.2	25	V	intra	Figure 6D	Taschner et al 2021
NSE4	268	NSE4	333	48.9	25	V	intra	Figure 6D	Taschner et al 2021
NSE4	268	NSE4	363	60.3	25	V	intra	Figure 6D	Taschner et al 2021
SMC5	235	SMC5	915	28.3	30	S	intra	Figure 6E	Yu et al 2021
SMC5	235	SMC5	909	22.9	30	S	intra	Figure 6E	Yu et al 2021
SMC5	248	SMC5	260	18.5	30	S	intra	Figure 6E	Yu et al 2021
SMC5	248	SMC5	259	16.7	30	S	intra	Figure 6E	Yu et al 2021
SMC5	227	SMC5	235	12.7	30	S	intra	Figure 6E	Yu et al 2021
SMC5	252	SMC5	260	12.5	30	S	intra	Figure 6E	Yu et al 2021

SMC5	111	SMC5	136	12.4	30	S	intra	Figure 6E	Yu et al 2021
SMC5	192	SMC5	980	11.7	30	S	intra	Figure 6E	Yu et al 2021
SMC5	252	SMC5	259	10.6	30	S	intra	Figure 6E	Yu et al 2021
SMC5	44	SMC5	136	10.2	30	S	intra	Figure 6E	Yu et al 2021
SMC5	971	SMC5	980	9.1	30	S	intra	Figure 6E	Yu et al 2021
SMC5	971	SMC5	979	6.7	30	S	intra	Figure 6E	Yu et al 2021
SMC5	245	SMC5	249	6.2	30	S	intra	Figure 6E	Yu et al 2021
SMC5	915	SMC5	919	6.1	30	S	intra	Figure 6E	Yu et al 2021
SMC5	245	SMC5	248	4.9	30	S	intra	Figure 6E	Yu et al 2021
SMC5	232	SMC5	235	4.7	30	S	intra	Figure 6E	Yu et al 2021
SMC6	288	SMC6	304	24.9	30	S	intra	Figure 6E	Yu et al 2021
SMC6	987	SMC6	1070	22.6	30	S	intra	Figure 6E	Yu et al 2021
SMC6	943	SMC6	958	22.6	30	S	intra	Figure 6E	Yu et al 2021
SMC6	989	SMC6	1059	18.8	30	S	intra	Figure 6E	Yu et al 2021
SMC6	987	SMC6	1067	18.1	30	S	intra	Figure 6E	Yu et al 2021
SMC6	1067	SMC6	1087	17.3	30	S	intra	Figure 6E	Yu et al 2021
SMC6	1059	SMC6	1070	17.1	30	S	intra	Figure 6E	Yu et al 2021
SMC6	987	SMC6	1059	15.3	30	S	intra	Figure 6E	Yu et al 2021
SMC6	1059	SMC6	1087	13.7	30	S	intra	Figure 6E	Yu et al 2021
SMC6	83	SMC6	160	13.4	30	S	intra	Figure 6E	Yu et al 2021
SMC6	1059	SMC6	1081	12.4	30	S	intra	Figure 6E	Yu et al 2021
SMC6	965	SMC6	996	12	30	S	intra	Figure 6E	Yu et al 2021
SMC6	1070	SMC6	1077	11.5	30	S	intra	Figure 6E	Yu et al 2021
SMC6	901	SMC6	906	11.2	30	S	intra	Figure 6E	Yu et al 2021
SMC6	958	SMC6	965	10.8	30	S	intra	Figure 6E	Yu et al 2021
SMC6	906	SMC6	913	10.7	30	S	intra	Figure 6E	Yu et al 2021
SMC6	1067	SMC6	1070	5.3	30	S	intra	Figure 6E	Yu et al 2021
NSE1	97	NSE1	247	18.2	30	S	intra	Figure 6F	Yu et al 2021
NSE1	99	NSE1	243	19.1	30	S	intra	Figure 6F	Yu et al 2021
NSE3	23	NSE3	66	14.2	25	S	intra	Figure 6F	Taschner et al 2021
NSE3	50	NSE3	66	15.8	25	S	intra	Figure 6F	Taschner et al 2021
NSE3	50	NSE3	122	9.5	25	S	intra	Figure 6F	Taschner et al 2021
NSE3	50	NSE3	236	17.1	25	S	intra	Figure 6F	Taschner et al 2021
NSE3	53	NSE3	66	12.6	25	S	intra	Figure 6F	Taschner et al 2021
NSE3	66	NSE3	122	20.7	25	S	intra	Figure 6F	Taschner et al 2021
NSE3	122	NSE3	236	14.2	25	S	intra	Figure 6F	Taschner et al 2021
NSE3	133	NSE3	283	13.6	25	S	intra	Figure 6F	Taschner et al 2021
